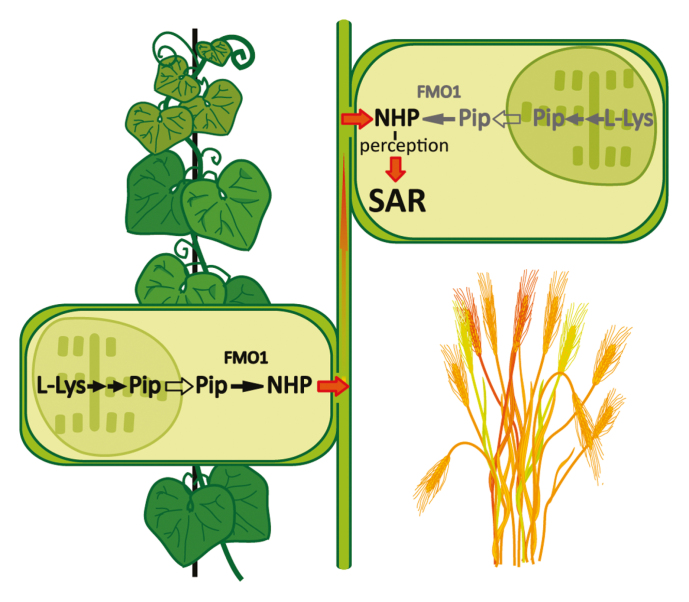# *N*-hydroxypipecolic acid: a general and conserved activator of systemic plant immunity

**DOI:** 10.1093/jxb/eraa345

**Published:** 2020-08-11

**Authors:** Tiziana Guerra, Tina Romeis

**Affiliations:** 1 Leibniz Institute of Vegetables and Ornamental Crops, Großbeeren, Germany; 2 Leibniz Institute of Plant Biochemistry, Halle (Saale), Germany

**Keywords:** *N*-Hydroxpipecolic acid, pipecolic acid, systemic acquired resistance, systemic immune signalling

## Abstract

This article comments on:

**Schnake A, Hartmann M, Schreiber S, Malik J, Brahmann L, Yildiz I, von Dahlen J, Rose LE, Schaffrath U, Zeier J**. 2020. Inducible biosynthesis and immune function of the systemic acquired resistance inducer *N*-hydroxypipecolic acid in monocotyledonous and dicotyledonous plants. Journal of Experimental Botany **71**, 6444–6459.

**Long-lasting and broad-spectrum disease resistance throughout plants is an ever-important objective in basic and applied plant and crop research. While the recent identification of *N*-hydroxpipecolic acid (NHP) and its central role in systemic plant immunity in the model *Arabidopsis thaliana* provides a conceptual framework toward this goal, Schnake *et al.* (2020) quantify levels of NHP and its direct precursor in six mono- and dicotyledonous plant species subsequent to attacks by their natural pathogens, thereby implicating (phloem-mobile) NHP as a general and conserved activator of disease resistance.**

## *N*-hydroxypipecolic acid in systemic acquired resistance

Systemic acquired resistance (SAR) denotes the establishment of an immune memory in plant parts distal from the local site of pathogen attack. The perception of pathogens in a primary local infection event induces immune signalling that activates local defences and is propagated to distal plant parts to prepare for future threats. In these so-called ‘primed’ leaves, a faster and stronger immune response is mounted when exposed to a subsequent pathogen attack, and leaf infection damage is reduced to a minimum (Navarova *et al.*, 2012; Hake and Romeis, 2019).

The nature of the systemic signalling molecules enabling the onset of priming and SAR is a long-standing matter of debate. Several metabolites have been discussed, which may propagate the information as a defence signal throughout the entire plant (Fu and Dong, 2013; Shah and Zeier, 2013). A recent hallmark study in Arabidopsis identified *N*-hydroxypipecolic acid (NHP) as a key molecule, which not only is induced by pathogen attack and accumulates at 24 h post-local infection in distal leaves, but is additionally sufficient to trigger (systemic) defence responses and disease resistance (Chen *et al.*, 2018; Hartmann *et al.*, 2018). NHP biosynthesis occurs in a three-step metabolic sequence from l-Lys through enzymes ALD1 (*AGD2-LIKE DEFENSE RESPONSE PROTEIN1*), SARD4 (*SAR-DEFICIENT4*), and FMO1 (*FLAVIN-DEPENDENT-MONOOXYGENASE1*). In particular, the last *N*-hydroxylation step catalysed by FMO1 has received a lot of attention, in which the precursor substrate pipecolic acid (Pip), demonstrated to be an immune modulator by itself (Navarova *et al.*, 2012), is converted to NHP. *FMO1* has independently been identified and characterized in Arabidopsis as required and sufficient to induce pathogen resistance (Bartsch *et al.*, 2006; Koch *et al.*, 2006; Mishina and Zeier, 2006). The ectopic expression of the *FMO1* gene resulted in reduced pathogen growth, and external application of NHP to leaves reduced systemic pathogen growth and potentiated SAR to bacterial and oomycete infections (Hartmann and Zeier, 2019). However, the complementary analysis of biochemical quantification of Pip and NHP metabolite levels and their dynamic kinetics during SAR induction was lagging behind for a long time because of the technical challenges.

## NHP biosynthesis and resistance function of NHP in mono- and dicotyledonous plants

Schnake *et al.* (2020) established six different natural plant pathosystems encompassing members of the dicotyledonous plant families Cucurbitaceae [cucumber (*Cucumis sativus*)–*Pseudomonas syringae* pv. *lachrymans*], Solanaceae [tomato (*Solanum lycopersicum*)–*Phytophthora infestans* or *P. syringae* pv. tomato]; tobacco (*Nicotiana tabacum*)– *P. syringae* pv. *tabaci*], and Fabaceae [soybean (*Glycine max*)–*P. savanastoi* pv. *glycinea*], and of the monocotyledonous Poaceae family [Brachipodium (*Brachypodium distachyon*)–*Xanthomonas translucens*, *Magnaporte oryzae* and barley (*Hordeum vulgare*)–*M. oryzae*]. In these bacterial and fungal plant pathosystems, metabolite levels of NHP, Pip, and the SAR-associated phytohormone salicylic acid (SA) were absolutely quantified over respective time courses after infections. In all investigated unstressed plant systems, basal free NHP levels were low or not detectable, whereas pathogen infection resulted in a 2- to 10-fold accumulation of NHP levels. Interestingly, SA increased upon bacterial infection in all investigated plant systems, but remained low upon fungal attacks in monocotyledonous Brachypodium and barley, not mirroring NHP induction.

Box 1. **Plant immunity through systemic acquired resistance (SAR)**

Plant immunity consists of several layers of defence that guarantee an interaction-specific blocking of respective intruders.

Plants memorize previous local attacks and prepare themselves against subsequent microbial infections either by the same or by different pathogens. This so-called systemic acquired resistance (SAR) requires the generation and propagation of a defence signal from the initial infected site to distal plant parts.

While the signalling cascades involved in local perception and downstream induction of defence responses are well studied, components mediating systemic signalling and their distal receptors are largely unknown.

*N*-Hydroxypipecolic acid (NHP) has recently been identified to be crucial for the induction and maintenance of SAR, and it is a matter of debate whether NHP may even serve as the key systemic signal itself. This concept is supported by research included herein showing that NHP is bioactive in resistance priming in mono- and dicotyledonous plants to infection by (hemi-) biotrophic bacterial, oomycete, and fungal pathogens.

These results extend data from a recent report on endogenous NHP production in different plant species upon treatment with a single bacterial pathogen *P. syringae* pv. tomato strain DC3000 (Holmes *et al.*, 2019). Furthermore, the external application of 1 mM NHP to leaves of cucumber, tobacco, Brachypodium, and barley triggered acquired resistance in these plants, and fewer disease symptoms became evident upon subsequent infections with the respective plant-specific pathogens. Thus, Schnake *et al.* (2020) document an overall presence of the NHP pathway and show that NHP potentiates acquired resistance in these plant systems. Remarkably, exemplified by the cucumber model, *P. syringae* pv. *lachrymans* induced high concentrations of NHP in both the local infected and the systemic uninoculated leaves, and in the respective local and systemic petiole exudates. These data provide strong evidence for vascular transport of NHP.

## Plant-specific conversion kinetics of Pip to NHP?

Detailed metabolite quantification in Schnake *et al.* (2020) reveals that in most investigated systems, NHP synthesis was paralleled by an increase in its direct precursor Pip, whereas the absolute levels of Pip to NHP vary between different species and pathosystems. In Solanaceae, Pip and NHP levels increase in parallel upon pathogen exposure, with final concentrations of Pip and NHP reaching either about the same level [tobacco ~6 μg g^–1^ FW at 72 h post-infection (hpi)] or with Pip exceeding NHP levels by ~8-fold (tomato). In Brachypodium and barley, fungal pathogen-induced Pip levels are up to 20- to 40-fold higher (barley 30 μg g^–1^ FW at 5 dpi) than those of NHP (0.1 μg g^–1^ FW), indicating that only small proportions of Pip are converted to NHP in these monocotyledonous plants. In contrast, cucumber displays constitutively high amounts of Pip already in both unstressed leaves (10 μg g^–1^ FW) and phloem sap. *Pseudomonas syringae* pv. *lachrymans* infection does not yield any further increase of Pip, whereas it nevertheless triggers accumulation of NHP (1.5 μg g^–1^ FW at 5 dpi). These data indicate that the mode and rate of the conversion from Pip to NHP are subject to a plant species-specific control. Whereas precursor Pip is present and stored in high amounts ready for its conversion to NHP in some species, the precursor will have to be synthesized *de novo* on demand in others.

Despite acknowledging distinct infection procedures [(spray-) inoculation, infiltration, or incubation], which may affect the total values measured in different pathosystems, an overall conserved pattern of pathogen-induced accumulation of (free) NHP seems to exist in different plant species. These data hint toward NHP as a general dose-dependent information mediator that triggers systemic signalling cascades to mount plant immunity.

In cucumber, with constitutive Pip levels measured, NHP biosynthesis may only underlie a strict control through the converting FMO1 enzyme as gatekeeper. In tobacco or tomato, in addition, Pip synthesis is likewise pathogen induced and depends on the activities of the biosynthesis enzymes ALD1 and SARD4, and on an export step out of the chloroplast organelle.

These data validate once more the crucial function of the FMO1 enzyme. Intensive phylogenetic studies highlight the conservation of the *FMO1* gene throughout the plant kingdom (Hartmann *et al.*, 2018; Holmes *et al.*, 2019). Ectopically expressed selected homologues of Arabidopsis FMO1 from different plant species catalyse the conversion from Pip to NHP, and metabolic engineering of NHP synthesis based on the Arabidopsis enzymes triggered SAR in tomato (Holmes *et al.*, 2019). Taken together with results from Schnake *et al.* (2020), these data not only provide the proof for *FMO1* as a valuable biotechnological target to engineer plant health and crop disease resistance by speeding up SAR signalling, but they also lead the way to investigate FMO1 enzymes from plant species other than Arabidopsis. It will be exciting to uncover their individual biochemical properties and enzyme kinetic parameters that help fulfil their function in the context of gatekeepers for NHP synthesis.

Box 2. **Stepping forward on NHP as a general activator of SAR**

Based on Schnake *et al.* (2020), the plant biology field will impatiently await future results addressing the following aspects:

(i)  What are the functional relationships between local and systemic versus phloem-mobile NHP? Pathogen infection triggers NHP *de novo* synthesis in local and systemic leaves, and NHP was shown to be phloem mobile. This raises the question about the origin and kinetics of leaf to phloem (mobile) free NHP linked to their function as a SAR activator.
(ii)  What is the role of NHP conjugates? While free NHP has been shown to suffice for the induction of local resistance and SAR in different plant species (Holmes *et al.*, 2019; Schnake *et al.*, 2020), a defined role for modified (e.g. glycosylated) NHP variants is as yet unclear. NHP conjugates may affect the concentration of an active component and may be correlated with the duration of immune signal and SAR memory.
(iii) How is NHP transported in and from the vasculature? The detection of substantial concentrations of NHP in the phloem sap of local and systemic leaves implicates transport processes for NHP from the cellular origin of synthesis into and out of the vasculature.
(iv) How is NHP perceived and translated to trigger immunity? Intensive genetic analyses have uncovered several upstream components required for NHP biosynthesis in local and distal leaves (e.g. for the expression and translation of biosynthesis genes *ALD1* or *FMO1*) and SAR, including *SARD1*/*CBP60g* and *PAD4*/*EDS1* (Hartmann *et al.*, 2018; Sun *et al*., 2018, 2020; Guerra *et al.*, 2020). In contrast, the mechanism of NHP perception and the nature of an NHP receptor still remain to be elucidated, though one candidate, NPR1 (*NONEXPRESSOR OF PR GENES 1*), has already been put forward for discussion (Hartmann and Zeier, 2019).